# MicroRNAs in Liver Cirrhosis: Diagnostic and Therapeutic Perspectives—A Comprehensive Review

**DOI:** 10.3390/jpm15080376

**Published:** 2025-08-14

**Authors:** Cristian Ichim, Adrian Boicean, Paula Anderco, Samuel Bogdan Todor, Adrian Hașegan, Sabrina Bîrsan, Victoria Bîrluțiu

**Affiliations:** Faculty of Medicine, Lucian Blaga University of Sibiu, 550024 Sibiu, Romania; cristian.ichim@ulbsibiu.ro (C.I.); samuelbogdant@gmail.com (S.B.T.); adrian.hasegan@ulbsibiu.ro (A.H.); sabrinaandreea.marinca@ulbsibiu.ro (S.B.); victoria.birlutiu@ulbsibiu.ro (V.B.)

**Keywords:** miRNAs, liver cirrhosis, hepatic stellate cells, fibrogenesis, biomarkers

## Abstract

Liver cirrhosis represents the end-stage of chronic hepatic injury, arising from a diverse range of etiologies including viral hepatitis, alcohol abuse and non-alcoholic fatty liver disease. A key driver of cirrhosis is hepatic fibrogenesis, a multifaceted process involving hepatic stellate cell activation, inflammatory signaling and extracellular matrix accumulation. MicroRNAs (miRNAs), a class of small non-coding RNAs, have emerged as pivotal regulators in this context, modulating gene expression networks that govern inflammation, fibrosis and hepatocarcinogenesis. This review synthesizes current evidence on the role of miRNAs in liver cirrhosis, emphasizing specific miRNAs such as miR-21, miR-122, miR-125, miR-146 and miR-155. These miRNAs influence pathways involving TGF-β, NF-κB and PI3K/Akt signaling, contributing to either fibrogenic progression or its suppression. The unique expression profiles and stability of miRNAs in biological fluids position them as promising non-invasive biomarkers for cirrhosis diagnosis and monitoring. Moreover, therapeutic modulation of miRNA activity through mimics or inhibitors holds future potential, though delivery and safety challenges remain. Advancing our understanding of miRNA-mediated regulation in cirrhosis could transform current diagnostic and therapeutic strategies, enabling more precise and personalized liver disease management.

## 1. Introduction

Cirrhosis marks the terminal stage of progressive liver injury and reflects a shared histopathological outcome across numerous chronic hepatic conditions [1,2,3]. Its underlying causes vary geographically: in Western countries, the most frequent triggers include chronic alcohol abuse, persistent hepatitis C virus infection and non-alcoholic fatty liver disease, while in the Asia-Pacific region, hepatitis B virus (HBV) remains the primary contributor to cirrhosis development [4,5,6,7,8]. In addition to these common etiologies, cirrhosis can also develop in the context of hereditary disorders, such as hemochromatosis and Wilson’s disease, or immune-related cholangiopathies, including primary biliary cholangitis, primary sclerosing cholangitis and autoimmune hepatitis [9,10,11,12,13].

MicroRNAs (miRNAs) represent a conserved class of small, endogenous non-coding RNA molecules, typically consisting of 19 to 25 nucleotides, which exert post-transcriptional regulation of gene expression [14]. Their existence remained entirely unrecognized until 1993, when a seminal study led by Victor Ambros and his team identified the first miRNA, marking a paradigm shift in the field of molecular biology [15,16]. This discovery catalyzed an exponential expansion of research, culminating in the identification of over 15,000 miRNAs across a broad spectrum of organisms, including plants, animals and viruses [17]. Since their initial characterization, miRNAs have been increasingly acknowledged as critical modulators of diverse cellular and molecular processes [18]. They have been implicated in the pathogenesis and progression of numerous pathological conditions, notably oncogenesis, neurodegenerative disorders such as Alzheimer’s and Parkinson’s disease, cardiovascular dysfunctions, chronic inflammatory states, hepatic disorders and obstetric complications including pre-eclampsia [17,19].

To date, over 1800 human miRNAs have been identified [20]. Computational analyses suggest that these regulate more than 60% of protein-coding genes through over 45,000 predicted target sites in the genome [21]. Given that a single miRNA can modulate entire gene networks, numerous miRNAs are implicated in key cellular functions and various liver pathologies, including viral hepatitis, steatohepatitis, fibrosis, cirrhosis and hepatocellular carcinoma (HCC) [22,23]. Owing to their remarkable stability in biological fluids, miRNAs have gained attention as promising non-invasive biomarkers for liver disease diagnosis and monitoring [24]. Recent evidence highlights that circulating miRNAs exhibit exceptional stability in plasma, serum, urine and saliva due to their encapsulation in exosomes, association with RNA-binding proteins (e.g., Argonaute2) or inclusion within high-density lipoproteins, which protect them from RNase-mediated degradation [25,26]. These properties allow miRNAs to withstand extreme pH variations, freeze–thaw cycles and prolonged storage, supporting their application as robust non-invasive biomarkers for liver disease diagnosis and monitoring [25,26,27].

miRNAs modulate gene expression programs across nearly all cell types and biological processes, including hepatic functions [28,29]. Dysregulated intracellular miRNA expression has been associated with a wide spectrum of liver diseases, including viral hepatitis, alcoholic and non-alcoholic steatohepatitis, drug-induced liver injury, autoimmune hepatitis and ischemia–reperfusion injury, with emerging evidence indicating that miRNA expression profiles vary significantly depending on the underlying disease etiology [29].

This review outlines current insights into miRNA involvement in liver cirrhosis, emphasizing key miRNAs with specific roles in disease progression. Additionally, it explores the emerging potential of miRNAs as diagnostic biomarkers and therapeutic targets in hepatic disorders.

## 2. Materials and Methods

This narrative review was developed based on a structured literature search aimed at identifying current evidence regarding the role of miRNAs in liver cirrhosis. The search was conducted across two major electronic databases: PubMed and Google Scholar, to ensure comprehensive coverage of the available scientific literature up to June 2025.

The search strategy employed combinations of keywords and Boolean operators, focusing on terms such as “microRNA”, “liver fibrosis”, “cirrhosis”, “hepatic stellate cells”, “hepatocellular carcinoma”, “chronic liver disease”, “biomarker” and “therapeutic target”.

Only peer-reviewed articles published in English were considered. Emphasis was placed on original research and review articles that investigated the expression, regulation or functional role of specific miRNAs in the context of hepatic fibrogenesis, cirrhosis or related liver pathologies. Following an initial screening of titles and abstracts, full-text articles were assessed for eligibility. The selection process prioritized studies offering mechanistic insights into miRNA regulation and function, as well as those reporting expression patterns in human or experimental liver disease models.

A total of 130 articles published until June 2025 were included after screening. Exclusion criteria comprised studies not focused on miRNA expression in hepatic disease models, those lacking mechanistic or translational relevance, non-peer-reviewed reports, conference abstracts and articles not available in English. Priority was given to studies providing experimental or clinical evidence of miRNA regulation and function in hepatic stellate cells, endothelial cells or other relevant liver cell populations.

## 3. Pathogenesis of Liver Cirrhosis

Chronic liver diseases arise from repeated hepatic injury triggered by viral infections (notably hepatitis B and C), metabolic toxins such as alcohol or drugs and autoimmune mechanisms [30,31]. In response to injury, hepatocyte damage initiates an inflammatory cascade, where Kupffer cells release cytokines and soluble mediators that activate hepatic stellate cells (HSCs), the principal effector cells in fibrogenesis [32,33,34]. Once activated, HSCs transform from quiescent vitamin-A-storing cells into contractile, extracellular-matrix-producing myofibroblasts, secreting profibrotic mediators including TGF-β1, PDGF and CTGF.

Recent studies highlight the dynamic recruitment of circulating monocytes to the injured liver, where they differentiate into proinflammatory macrophages, amplifying immune cell infiltration and perpetuating tissue injury [35]. In a murine model of 70% hepatectomy, Ly6C^+^CD11b^+^ monocytes/macrophages infiltrated the regenerating liver, accompanied by a reduction in CD206^+^ and CD163^+^ resident macrophages, indicating a phenotypic shift within the hepatic macrophage population [35]. Transcriptomic and microRNA profiling of these cells revealed altered expression of inflammation-related pathways and microRNAs involved in cell cycle control and apoptosis, underscoring the contribution of macrophage plasticity and microRNA-driven regulatory networks to liver remodeling and immune activation in injury contexts, including cirrhosis.

Multiple microRNAs (e.g., miR-29, miR-21, miR-155) tightly regulate HSC activation and endothelial cell phenotype, influencing extracellular matrix remodeling and fibrogenic signaling cascades [36,37,38]. Excessive extracellular matrix deposition creates an imbalance between fibrogenesis and fibrolysis, resulting in scar formation, architectural disruption and ultimately cirrhosis [39]. As fibrosis progresses from bridging fibrotic bands to nodular regeneration, liver function declines, potentially culminating in failure and high mortality rates [40].

Hepatic fibrogenesis is orchestrated by a complex interplay of cytokines, growth factors, vasoactive mediators and adipokines, each contributing to the balance between fibrosis progression and resolution [41]. Proinflammatory cytokines such as monocyte chemotactic protein-1 and RANTES have been shown to stimulate fibrogenic responses, whereas interleukin-10 (IL-10) and interferon-γ exert inhibitory effects on fibrosis development [42,43,44].

Among growth factors, transforming growth factor-β1 (TGF-β1) is a key profibrotic mediator in humans; it promotes HSC transdifferentiation into myofibroblast-like cells, enhances extracellular matrix production and reduces its degradation [45,46]. TGF-β1 also regulates MMPs and their inhibitors, influencing matrix remodeling and immune cell function and experimental strategies targeting this signaling pathway have led to significant reductions in fibrosis [45,46].

Platelet-derived growth factor (PDGF) is another major profibrotic factor, recognized as the most potent mitogen for HSCs. Its expression is upregulated in the fibrotic liver and its inhibition results in attenuation of experimental fibrogenesis [47].

Vasoactive substances also modulate fibrotic outcomes: vasodilators like nitric oxide and relaxin exhibit antifibrotic properties, while vasoconstrictors such as norepinephrine, angiotensin II and endothelin-1 drive fibrosis [48,49,50]. Angiotensin II, the primary effector of the renin–angiotensin system (RAS), plays a central role in fibrogenesis by inducing HSC proliferation, cytokine secretion and collagen synthesis [51]. These effects are largely mediated through reactive oxygen species generated by non-phagocytic nicotinamide adenine dinucleotide phosphate (NADPH) oxidase, which are constitutively expressed in fibrogenic cell types and further activated by inflammatory stimuli [52,53,54,55,56]. Genetic or pharmacological inhibition of RAS markedly attenuates fibrosis in experimental models and disruption of NADPH oxidase protects against alcohol- and bile-duct-ligation-induced liver injury [53,57,58,59].

Adipokines, secreted predominantly by adipose tissue, provide a metabolic link to liver fibrosis. Leptin is essential for HSC activation and fibrosis progression, whereas adiponectin demonstrates robust antifibrotic effects both in vitro and in vivo [30,60,61]. These opposing roles may partly explain the association between obesity and increased fibrosis risk in chronic hepatitis C [41].

Advances in understanding the mechanisms of liver fibrogenesis, including cytokine signaling, HSC activation and extracellular matrix turnover, have led to effective antifibrotic interventions in experimental models [31,32]. However, reversing established fibrosis remains a major therapeutic challenge, as many patients present only at advanced stages. Current treatments targeting inflammation offer limited efficacy in late-stage disease, where liver transplantation remains the only curative option, although its success is constrained by limited organ availability [33]. These limitations underscore the need for novel strategies that modulate gene expression to treat or reverse liver fibrosis and cirrhosis. Thus, liver cirrhosis arises as the end-stage consequence of chronic liver injury, with fibrosis representing a key intermediate phase [34].

## 4. Specific miRNAs Involved in Liver Diseases

miRNAs play a crucial role in modulating HSC activation and thus influence the progression of liver fibrosis. In rats, downregulation of miR-27a and miR-27b promotes a quiescent HSC phenotype, marked by lipid droplet accumulation and decreased proliferation [62]. miR-29b acts as a negative regulator of type I collagen and SP1 in HSCs and has been shown to modulate liver fibrosis via TGF-β1 and NF-κB signaling in both human and murine models [63,64]. As such, the miR-29 family has emerged as a key antifibrotic regulator.

miRNA profiling in CCl_4_-induced liver fibrosis and human samples identified miR-199a, miR-199a, miR-200a and miR-200b* as significantly upregulated during fibrosis progression [65]. These miRNAs contribute to fibrogenesis through distinct yet converging pathways: miR-199a and miR-200b* modulate the TGF-β signaling axis by targeting components such as SMURF2, while members of the miR-200 family additionally regulate epithelial-to-mesenchymal transition via suppression of ZEB1 and SIP1 [65].

Similarly, miR-214-5p enhances the expression of profibrotic markers including MMP-2, MMP-9, α-SMA and TGF-β1 in LX-2 cells [66]. In parallel, miR-221 and miR-222 exhibit stage-dependent upregulation in both human liver tissue and murine models of fibrosis. These are induced by TGF-α and TNF-α, with miR-222 shown to directly suppress CDKN1B (p27) via 3′-UTR binding [67]. Collectively, the overexpression of these miRNAs correlates strongly with fibrosis severity and promotes hepatic stellate cell activation and extracellular matrix production, reinforcing their central role in the orchestration of fibrogenic signaling cascades [65].

Several antifibrotic miRNAs, including miR-150 and miR-194, are downregulated in HSCs during liver fibrosis, where they normally inhibit HSC activation and extracellular matrix production by targeting c-myb and rac1 [68]. Similarly, miR-29, miR-19b, miR-146a and miR-133a are also suppressed in fibrotic models and their restoration has been shown to mitigate fibrogenesis [64,69,70]. These data underscore the potential of specific miRNAs as both biomarkers of HSC activation and therapeutic targets in liver fibrosis and cirrhosis.

Recent findings suggest compartment-specific roles for miR-571 and miR-652 in liver cirrhosis [71]. miR-571 is significantly upregulated in the serum of patients with advanced cirrhosis (Child–Pugh C) and correlates with disease severity. Induced by TGF-β in hepatocytes and hepatic stellate cells (HSCs), miR-571 promotes fibrogenesis by increasing α-SMA and Col1A1 expression and downregulating CREBBP [71]. Its parallel upregulation in both serum and liver tissue supports its potential as a fibrosis-associated biomarker and therapeutic target. In contrast, miR-652 is downregulated in the serum independent of cirrhosis stage [71]. This decrease is attributed to reduced expression in circulating monocytes, driven by proinflammatory stimuli, suggesting its involvement in systemic immune regulation rather than direct hepatic fibrogenesis.

Several microRNAs have been identified as potential diagnostic and prognostic markers in extrahepatic cholangiocarcinoma, reflecting their diverse roles in tumor biology [72]. miR-145 functions as a tumor suppressor and is markedly downregulated in extrahepatic cholangiocarcinoma, with decreased expression linked to increased tumor proliferation, invasion and adverse clinical outcomes [73]. Similarly, members of the miR-200 family, which regulate epithelial–mesenchymal transition, are commonly suppressed in cholangiocarcinoma, facilitating cellular migration and metastasis [72,74].

### 4.1. a. miR-21

miR-21, encoded on chromosome 17q23.2, is among the most prevalent circulating microRNAs and exhibits broad tissue distribution, being highly expressed in the bone marrow, liver, lung, kidney, gastrointestinal tract and thyroid gland [38,75]. At the cellular level, it localizes to the cytoplasm and is also enriched in extracellular exosomes [76,77]. Functionally, miR-21 plays a pivotal role in the regulation of inflammation, fibrogenesis and tumorigenesis and is notably overexpressed in liver malignancies [78,79]. It has also been found to be significantly upregulated in individuals with overweight or obesity, showing a strong association with elevated body mass index and increased total cholesterol levels [80]. miR-21 is consistently elevated in the serum of patients with cholangiocarcinoma and has been identified as an oncogenic miRNA associated with tumor progression and poor prognosis [72].

### 4.2. b. miR-122

miR-122, located on chromosome 18, is the most abundant hepatic microRNA and exerts essential regulatory functions in liver physiology [81]. It is involved in metabolic homeostasis, particularly in the regulation of fatty acid and cholesterol metabolism and contributes to liver development through its roles in hepatocyte proliferation, differentiation, maturation and polyploidy [82,83,84,85]. Antisense inhibition of miR-122 has been shown to lower plasma cholesterol levels in both mice and chimpanzees [86,87,88].

**Figure 1:**
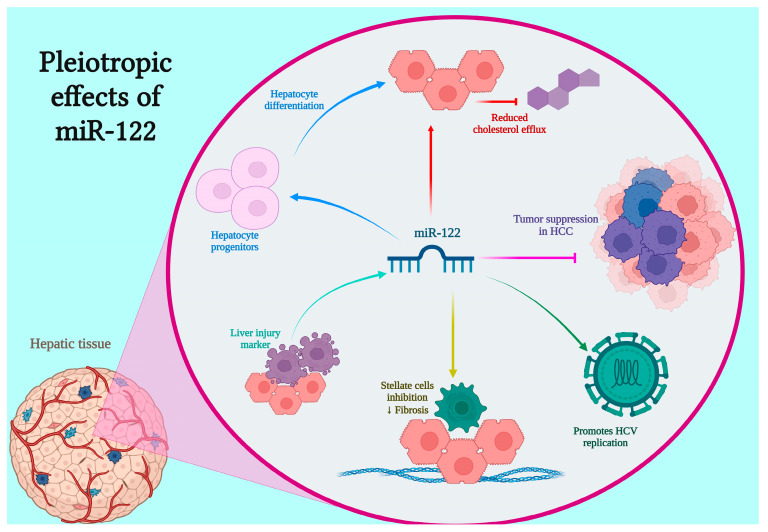
Pleiotropic effects of miR-122 in hepatic tissue. miR-122, a liver-specific microRNA, exerts diverse regulatory functions in liver physiology and pathology. It promotes hepatocyte differentiation from progenitor cells (blue), suppresses hepatocellular carcinoma (HCC) progression (purple), inhibits hepatic stellate cell activation and fibrosis (yellow) and serves as a sensitive marker of liver injury (aqua green). Conversely, miR-122 supports hepatitis C virus (HCV) replication (green) and suppresses cholesterol efflux, influencing lipid metabolism (red).

Loss of miR-122 promotes fibrogenesis and tumorigenesis by disrupting cell cycle control and key oncogenic pathways [89]. Importantly, miR-122 has emerged as both a diagnostic biomarker and a potential therapeutic target, with therapeutic restoration strategies under investigation for chronic liver disease and HCC [89]. Reduced hepatic miR-122 expression has also been observed in human non-alcoholic steatohepatitis, corresponding animal models and in murine models of alcoholic liver disease [90,91,92]. Notably, genetic deletion of miR-122 in mice induces progressive liver damage, including steatohepatitis, fibrosis and HCC [93,94]. Its expression is tightly controlled by liver-specific transcription factors, including C/EBP, HNF1, HNF3 and HNF4 [82]. Given its release during inflammatory processes such as viral infections and liver cancer, miR-122 is also considered a promising biomarker for early detection of liver injury [95,96] (Figure 1).

### 4.3. c. miR-125

In RKO colorectal cancer cells, miR-125 functions as a tumor suppressor by directly targeting and inhibiting the expression of vascular endothelial growth factor [97]. Overexpression of miR-125 leads to reduced cell viability, proliferation (evidenced by decreased PCNA levels) and migration, while simultaneously increasing apoptosis, as shown by enhanced caspase-3 activity and DNA fragmentation [97]. Furthermore, miR-125 suppresses the MAPK signaling pathway by downregulating phosphorylated forms of ERK, p38 and JNK [97,98]. Additionally, miR-125 decreases COX-2 expression, supporting its involvement in inflammatory regulation in colorectal cancer [97,99].

In HCC, miR-125 acts as a tumor suppressor by directly targeting the oncogene Pokemon (Zbtb7) [100]. Its downregulation promotes tumor growth, while miR-125 overexpression inhibits proliferation and invasion. Moreover, a negative feedback loop exists, whereby Pokemon represses miR-125 transcription, establishing a self-sustaining circuit that contributes to HCC progression [100].

### 4.4. d. miR-146

miR-146a and miR-146b act as protective regulators in small-for-size liver graft injury by suppressing key inflammatory mediators [101]. Specifically, both miRNAs were downregulated after liver reperfusion, leading to increased expression of IRAK1 and TRAF6, two targets in the TLR4/NF-κB signaling pathway [101]. Administration of miR-146a/b mimics significantly attenuates liver damage, lowering serum ALT/AST, TNF-α, IL-6 levels and histological injury [101]. These effects were mediated through direct inhibition of IRAK1 and TRAF6, thus dampening the inflammatory response and improving 21-day survival in rats.

miR-146a promotes HBV replication and antigen expression by directly targeting ZEB2, a transcriptional repressor of the HBV core promoter [102]. Its upregulation increases HBV DNA levels and viral antigen secretion, identifying miR-146a as a positive regulator of HBV replication [102].

miR-146a acts as a key negative regulator of inflammation by suppressing proinflammatory signaling pathways [103]. It targets IRAK1 and TRAF6, thereby modulating NF-κB activation and controlling the production of cytokines such as TNF-α and IL-8 [104]. Its upregulation contributes to immune tolerance, protects against sepsis-induced cardiac dysfunction and maintains barrier integrity in epithelial tissues [104,105,106]. miR-146b, similarly, dampens inflammatory responses, particularly in monocytes and endothelial cells, and is regulated by IL-10/STAT3 signaling [104]. Together, these miRNAs maintain immune homeostasis and prevent excessive inflammatory responses.

### 4.5. e. miR-155

miR-155 plays a multifaceted role in inflammation, immunity and oncogenesis [107,108]. Encoded by the BIC gene, miR-155 is induced by inflammatory stimuli such as TNF-α, IL-1β, interferons and TLR ligands [109]. It exerts context-dependent regulation by targeting transcripts involved in immune responses, including SHIP1, SOCS1 and TAB2 [109]. Both strands, miR-155-5p and miR-155-3p, contribute to immune modulation, sometimes acting antagonistically. Importantly, miR-155 has been implicated in over 60 pathological conditions, including lymphomas, asthma, cystic fibrosis and tuberculosis [110,111]. In these contexts, it regulates cytokine production, immune cell differentiation and fibrotic signaling pathways such as PI3K/Akt and TGF-β [109]. Its dysregulation is linked to disease progression and anti-miR-155 therapies are under clinical investigation.

miR-155 regulates immune and inflammatory pathways in liver disease, promoting cytokine expression, cell cycle disruption and proliferation [112]. It is involved in fibrosis, viral and metabolic liver diseases and HCC, with potential as both a biomarker and therapeutic target [112].

In cancer biology, miR-155 has been implicated across a broad spectrum of malignancies, including breast, lung and colorectal cancers, as well as oral squamous cell carcinoma, cervical and pancreatic cancers, nasopharyngeal carcinoma and various forms of leukemia [113,114,115,116].

miR-155-5p has been shown to contribute to liver fibrosis and cirrhosis by promoting M1 macrophage polarization through direct targeting of SOCS1, which in turn activates the JAK1/STAT1 signaling pathway [117]. This cascade enhances hepatic lymphangiogenesis, a process tightly associated with disease progression [117]. Moreover, miR-155-5p levels are elevated in both liver tissue and peripheral blood in CCl_4_-induced murine models, reinforcing its potential as both a biomarker and therapeutic target in liver fibrosis.

Table 1 summarizes the most relevant miRNAs implicated in the processes of inflammation, fibrosis, liver regeneration and tumorigenesis in the context of chronic liver diseases.

## 5. Therapeutic Challenges and Progress in miRNA-Based Treatments

In the field of treatment, research progress has been even slower, with currently no approved drug utilizing microRNAs as a therapeutic basis for any disease. This is partly due to the fact that diagnosis and treatment evolve in parallel, with the latter progressing slowly without advancements in the former. Nevertheless, there remains hope that the future (despite increasingly challenging conditions for conducting clinical trials) will bring new, personalized and targeted therapies capable of curing diseases not only in the digestive sphere but also in related areas.

In the field of cancer therapy, clinical trials have tested microRNA-based drugs. One such trial, published in 2020, reported results in patients with hepatocellular carcinoma using microRNA-34a therapy, but the conclusions were not as expected [126]. Although some positive results were obtained in solid tumors, the trial was halted due to severe and uncontrollable immune-related adverse reactions. Despite prophylactic dexamethasone administration to mitigate risk, four patients died, leading to the discontinuation of the study [126]. Toxicity might be reduced by implementing delivery systems based on extracellular vesicles, which are preferentially taken up by the liver and could serve as optimal carriers for therapeutic delivery. The explanation for the high toxicity of current microRNA-based therapies lies in the inability to adequately control the specificity of particular microRNAs, as they often regulate a very large number of target genes, leading to numerous adverse reactions [127,128,129]. For this reason, no microRNA-based treatment has yet been officially approved and research is progressing with great difficulty.

## 6. Future Directions

While considerable progress has been made in elucidating the roles of microRNAs in liver cirrhosis, numerous gaps remain that warrant further investigation. Future studies should prioritize the validation of miRNA signatures in large, well-characterized patient cohorts, ideally incorporating multietiological origins of cirrhosis to ensure clinical relevance and applicability.

In particular, longitudinal analyses are needed to determine whether specific miRNA profiles can predict disease progression, therapeutic response or risk of decompensation and HCC. Integration of miRNA profiling with other omics technologies such as transcriptomics, proteomics and metabolomics may yield more comprehensive biomarker panels with enhanced diagnostic accuracy. The integration of artificial intelligence and machine learning approaches with multiomics datasets, including genomics, transcriptomics, proteomics and metabolomics, provides a promising avenue to develop predictive models for cirrhosis progression and risk stratification, surpassing traditional clinical scores; however, overcoming challenges in data heterogeneity, interpretability and model generalizability remains critical for clinical implementation.

On a mechanistic level, the cell-type-specific roles of miRNAs in the hepatic microenvironment remain incompletely understood. Studies using single-cell sequencing and spatial transcriptomics could help dissect the compartmentalized expression and function of miRNAs across hepatocytes, stellate cells, endothelial cells and immune populations. Moreover, the crosstalk between circulating and intracellular miRNAs in modulating fibrotic signaling cascades represents an emerging area of interest.

Therapeutically, the development of miRNA mimics or inhibitors (antagomirs) shows promise, but delivery strategies remain a major hurdle. Targeted delivery systems that ensure cell-specific uptake and minimize off-target effects are essential for clinical translation. Despite significant pre-clinical advances, miRNA-based therapeutics face critical challenges, including achieving cell-specific delivery, avoiding off-target effects, ensuring long-term stability and overcoming immune activation. Notably, no miRNA-targeted therapy has yet received regulatory approval, despite more than three decades of research, underscoring the translational gap that must be bridged before these platforms can impact cirrhosis care.

Future clinical trials incorporating miRNA-based diagnostics or therapeutics must evaluate not only efficacy but also long-term safety, cost-effectiveness and integration into existing care pathways.

## 7. Conclusions

Liver cirrhosis remains a global health challenge, representing the irreversible outcome of chronic hepatic injury driven by a complex interplay of cellular and molecular mechanisms. Among these, miRNAs have emerged as essential regulators of fibrosis and cirrhosis, modulating processes such as hepatic stellate cell activation, immune responses and extracellular matrix remodeling.

A growing body of evidence highlights the dual role of miRNAs as both pathogenic effectors and therapeutic targets, depending on their expression context and cellular origin. Several miRNAs, including miR-21, miR-122, miR-146 and miR-155, have demonstrated mechanistic relevance in pre-clinical models and show promise as non-invasive biomarkers and candidates for targeted therapy.

Despite these advances, significant challenges remain in translating basic research into clinical application. Continued interdisciplinary efforts are necessary to refine miRNA-based diagnostics and therapeutics, ultimately improving outcomes for patients with liver cirrhosis. The integration of miRNA knowledge into routine hepatology practice holds transformative potential, paving the way for more personalized, predictive and precise approaches to liver disease management. Future efforts should focus on bridging the gap between experimental data and real-world clinical tools to fully harness the promise of miRNA-guided hepatology.

## Figures and Tables

**Table 1:** Expression Profiles and Functional Roles of Selected MicroRNAs Involved in Liver Disease Pathogenesis.

miRNA	Expression in Liver Disease	Main Role	Target Genes	Liver Context	Source
**miR-155**	Upregulated in liver inflammation and fibrosis	Promotes inflammation and fibrogenesis via FOXO3a suppression	FOXO3a, SOCS1, TP53INP1, STAT3	Involved in hepatic stellate cell activation and fibrosis in CCl_4_-induced liver injury	[118,119,120,121]
**miR-155-5p**	Upregulated in HCC and liver inflammation	Promotes proliferation and inhibits apoptosis through PI3K/Akt signaling	PTEN, SOCS1	Overexpressed in HCC; enhances tumor growth and suppresses apoptotic signaling	[122]
**miR-125a-3p**	Upregulated during regeneration	Enhances hepatocyte proliferation and liver regeneration	PRAP1	Promotes liver regeneration post-hepatectomy	[123]
**miR-21**	Upregulated	Promotes fibrosis and tumor progression	Multiple fibrosis/cancer genes	Elevated in cirrhosis and HCC	[124]
**miR-146a**	Variable (often upregulated in inflammation)	Regulates immune response; NF-κB pathway feedback inhibitor	IRAK1, TRAF6	Implicated in sepsis-related liver inflammation and immune tolerance	[125]
**miR-148a-5p**	Downregulated in liver fibrosis	Inhibits HSC activation and fibrosis	SLIT3	Restored by human umbilical cord mesenchymal stem cell therapy; reduces fibrosis via HSC modulation	[121]

## Data Availability

No new data were created.
